# Electromicrofluidic Device for Interference-Free Rapid Antibiotic Susceptibility Testing of *Escherichia coli* from Real Samples

**DOI:** 10.3390/s23239314

**Published:** 2023-11-21

**Authors:** Sonal Fande, Khairunnisa Amreen, D. Sriram, Valentin Mateev, Sanket Goel

**Affiliations:** 1 MEMS, Microfluidic and Nanoelectronics Lab, Department of Electrical and Electronics Engineering, Birla Institute of Technology and Science, Hyderabad 50078, India; 2 Department of Pharmacy, Birla Institute of Technology and Science, Hyderabad 500078, India; 3 Department of Electrical and Electronics Engineering, Birla Institute of Technology and Science, Hyderabad 500078, India; 4 Department of Electrical Apparatus, Technical University of Sofia, 1156 Sofia, Bulgaria

**Keywords:** antibiotic susceptibility testing, microfluidic, antimicrobial resistance, *E. coli*, multidrug resistance, minimum inhibitory concentration

## Abstract

Antimicrobial resistance (AMR) is a global health threat, progressively emerging as a significant public health issue. Therefore, an antibiotic susceptibility study is a powerful method for combating antimicrobial resistance. Antibiotic susceptibility study collectively helps in evaluating both genotypic and phenotypic resistance. However, current traditional antibiotic susceptibility study methods are time-consuming, laborious, and expensive. Hence, there is a pressing need to develop simple, rapid, miniature, and affordable devices to prevent antimicrobial resistance. Herein, a miniaturized, user-friendly device for the electrochemical antibiotic susceptibility study of *Escherichia coli* (*E. coli*) has been developed. In contrast to the traditional methods, the designed device has the rapid sensing ability to screen different antibiotics simultaneously, reducing the overall time of diagnosis. Screen-printed electrodes with integrated miniaturized reservoirs with a thermostat were developed. The designed device proffers simultaneous incubator-free culturing and detects antibiotic susceptibility within 6 h, seven times faster than the conventional method. Four antibiotics, namely amoxicillin–clavulanic acid, ciprofloxacin, ofloxacin, and cefpodoxime, were tested against *E. coli*. Tap water and synthetic urine samples were also tested for antibiotic susceptibility. The results show that the device could be used for antibiotic resistance susceptibility testing against *E. coli* with four antibiotics within six hours. The developed rapid, low-cost, user-friendly device will aid in antibiotic screening applications, enable the patient to receive the appropriate treatment, and help to lower the risk of anti-microbial resistance.

## 1. Introduction

The accurate and early detection of microbial infection, followed by appropriate treatment via antibiotic administration, is pivotal in reducing the fatality and severity of the disease in a patient [1,2]. Antibiotics are effective against bacterial infection, by killing the bacteria or inhibiting its growth [3]. Alexander Fleming, a physics scientist, accidentally discovered the first antibiotic, penicillin, to treat bacterial infection. That simple discovery saved millions of lives over decades [4]. Since then, several antibiotics have been prepared and discovered over the years. However, with time, these microorganisms become resistant to drugs. Antimicrobial resistance arises when microbes do not have a more extended response to the medicine, making the infection harder to treat [5]. Therefore, with minimalistic symptoms and disease onset, it is crucial to identify the microorganism and the antibiotic effective against it. An antimicrobial resistance (AMR) test is often conducted; AMR shows the type and quantity of antibiotic working against the microorganism [6].

Various pathogens, like bacteria, fungi, viruses, and parasites, cause infection and form resistance [7]. Among these, bacterial and viral infections are more prevalent [8]. Pneumonia, diarrhea, and urinary tract infections are the most pervasive bacterial illnesses caused by *Escherichia coli* [9]. *E. coli* is the most known bacterium that causes multidrug resistance [10]. Improper use of antibiotics, multiple illnesses, and prolonged stays in the hospital are critical risk factors for *E. coli* multidrug resistance [11]. Therefore, knowing the antibiotic effect, dosage, and duration before use is essential. Antibiotic susceptibility testing (AST) helps identify the pathogen and the most effective antibiotic against it [12,13]. AST provides information on selecting antibiotics and evaluates the minimum inhibitory concentration. It detects both phenotype and genotype resistance. Genotype is classified based on the presence or absence of a resistant gene, and phenotype is found without the gene mutation. Different techniques are available; among them, disk diffusion is the gold standard for AST, as is quick to execute, can identify many antibiotics in a single test, and allows for a wide range of antibiotic choices. Still, it takes time and cannot provide minimum inhibitory concentration values. Another method is broth dilution [14], which is straightforward, legitimate, and easily accessible but demands more supplies of reagents and introduces more room for error. Moreover, these traditional AST approaches are time-consuming and labor-intensive, requiring skilled laboratory set-up and bulky instrumentation [15]. Often, a time frame of 4–5 days is reportedly needed to study the resistance clinically. Owing to this, the infection increases, and sometimes delays can even be fatal [16]. Hence, developing rapid techniques for measuring antibiotic effectiveness will improve global health and decrease mortality [17]. In this context, miniaturized and microfluidics-based devices provide possible solutions [18,19]. Microfluidic-based devices offer multiple advantages of reduced assay time, low cost, simple operation, and increased testing efficacy [20,21]. However, some microfluidic devices need high resolution. A microfluidic device has recently been designed to separate microbial cells using a ferrohydrodynamic approach. However, the developed device is complex and requires more attention to the environmental effect on fluid flow inside the channel [22]. Hence, microfluidic integrated with electrochemical sensing further improves the detection efficacy and increases the simplicity and sensitivity of detection [23,24].

**Figure 1:**
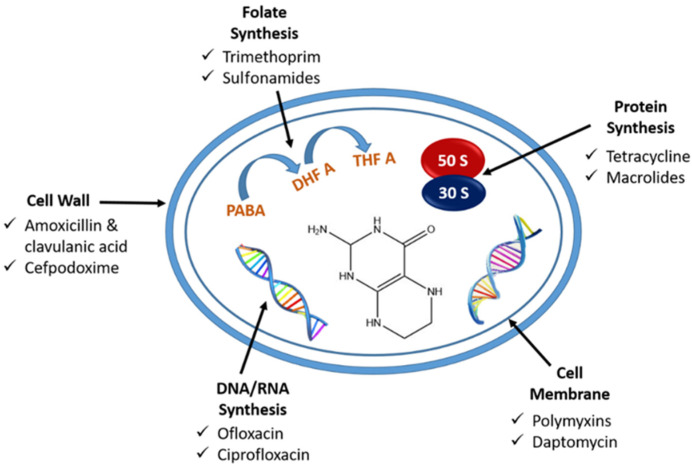
Schematic representation of the mechanism of action of antibacterial drugs.

The present study is an extension of our previous work [25]. Herein, we developed a rapid, sensitive, miniaturized electrochemical device for simultaneous culturing, detection, and antibiotic susceptibility study [26,27]. Here, *E. coli* was used as a model microbe for testing the device. A screen-printed electrode system modified with graphitized mesoporous carbon (GMC) was used for testing [28]. GMC is a high surface area carbon material that helps sensitively detect *E. coli* [29,30]. The microfluidic channel was designed and integrated with screen-printed electrodes. The in-house laser-induced graphene (LIG) heater was fabricated to incubate bacterial culture. Various antibiotics were screened by checking the minimum inhibitory zone, and the one with a more significant inhibition zone was selected for susceptibility testing. Different antibiotic concentrations were prepared, and efficacy was checked using the electrochemical cyclic voltammetry (CV) method. The specificity of antibiotics towards *E. coli* was validated using *Streptococcus pneumoniae*, *Pseudomonas aeruginosa*, and *Shewanella putrefaciens* bacteria. The real sample analysis was done using artificial urine and water samples. The obtained results were further validated with the conventional broth dilution method. To the extent feasible, this is a benchmarking prototype study that has yet to be explored further. The strategy with further optimizations can also be used for other microorganisms in real-time. Figure 1 shows the mechanism of action of different antibiotics to prevent bacterial growth.

## 2. Experimental Method

### 2.1. Materials

Luria broth and Luria agar were procured from Thermo Fisher Scientific, Delphi, India. Potassium chloride, carbon ink, glass slides (75 × 50 mm), ammonium phosphate, sodium sulfate, ammonium diphosphate, magnesium chloride, calcium chloride, creatinine, and urea were purchased from Sigma Aldrich, Ltd. (Burlington, MA, USA). *E. coli* culture was acquired from the Biological Science Department, BITS Pilani Hyderabad campus. Clavam 625 (amoxicillin and clavulanic acid), Zenflox 200 (ofloxacin), Monocef-O 200 (cefpodoxime), Cifran 500 (ciprofloxacin), and Azee-500 (azithromycin) were purchased from a local medical store. Polydimethylsiloxane (PDMS) was purchased from Delta Silicon, Mumbai, India. A CO_2_ laser (VLS 3.20) was procured from Universal Laser Systems, Scottsdale, AZ, USA.

### 2.2. Development of Three-Electrode System and Microfluidic Device

**Figure 2:**
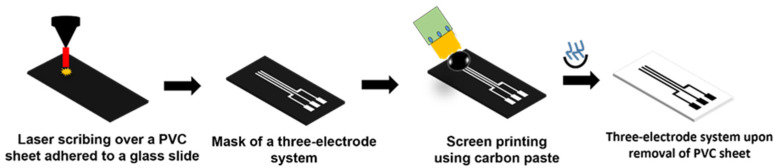
Schematic for the development of a three-electrode system using the screen-printing technique.

A three-electrode system was fabricated using the screen-printing technique. The design of the requisite dimension was first drawn on SolidWorks software. A polyvinyl chloride (PVC) sheet was attached to a glass side (75 × 50 mm), and the laser was scribed over the PVC sheet to prepare the mask. Carbon ink was laid down over the obtained mask with the help of a squeeze and kept in the oven for 30 min at 60 °C. The PVC sheet was removed after drying, and the screen-printed electrodes were obtained [31]. Figure 2 depicts a detailed schematic of the fabrication process.

**Figure 3:**
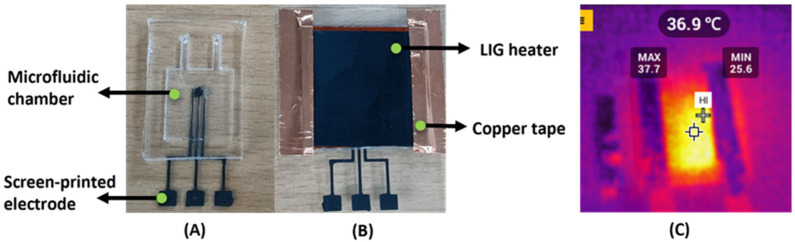
(**A**) A miniaturized device integrated with (**B**) a laser-induced graphene (LIG) heater provides temperature, and copper tape provides electrical contacts connected to the voltage regulator. (**C**) The LIG heater captured by a thermal camera after heating while providing a voltage of 2.5 V.

A mold (2 × 1.4 × 17 mm^3^) was prepared on an acrylic sheet to develop the microfluidic device. To create a PDMS mixture, epoxy and curing agent were mixed in a 10:1 ratio and degassed for 30 min to remove oxygen bubbles. Following this, PDMS was run over the mold and baked for an hour at 60 °C. Post-curing, the reservoir was cut from the mold and bonded over the three-electrode system using the plasma bonding method. The developed microfluidic device integrated with the laser-induced graphene (LIG) heater is shown in Figure 3. The details of the fabrication scheme for screen-printed electrodes, microfluidic device fabrication, and its integration, as well as how to prepare bacteria samples, were covered in greater depth in our previous research [25].

### 2.3. Fabrication of LIG Heater

For the fabrication of the LIG heater, a polyamide sheet of the required dimension of 25 × 25 mm was initially pasted onto a glass slide surface using double-sided tape. The CO_2_ laser (VLS 3.60) was exposed on the polyimide sheet with power and speed of 6.5% and 4.5% to obtain laser-induced graphene [32]. After engraving, the obtained thickness was 50 µm. Electrical contacts were provided on the fabricated LIG film using copper tape and silver ink. The thermal factor was calibrated earlier by varying the potential and noting the temperature. A temperature of 37 °C was maintained by applying a potential of 2.5 V. A thermal camera was used to keep track of the achieved temperature. Figure 3C shows the temperature of the LIG heater, which was maintained at 37 ± 10 °C.

### 2.4. Effect of Antibiotic on E. coli

Electrochemical analysis was used to determine the effect of antibiotics on bacterial growth. A Potentiostat (CHI 1030E) was utilized to record the electrochemical response. A three-electrode setup was employed, with a reference electrode of Ag/AgCl, a working electrode of GMC, and a counter electrode of plan carbon ink.

### 2.5. Evaluation of Real Samples

The real samples used for analysis were synthetic urine and tap water [14]. The synthetic urine was prepared by adding all dried components to sterile water. Normal urine is a mixture of organic compounds such as urea, creatinine, and uric acid and inorganic substances like ammonia, sulfates, chloride, and phosphates. The composition of prepared synthetic urine is provided in Table 1.

## 3. Results and Discussion

### 3.1. Off-Chip Minimum Inhibitory Concentration (MIC) Measurement

The MIC describes the resistance or susceptibility of the particular bacteria toward valuable antibiotics. The model microorganism used was *E. coli* (DH5α strain) for MIC calculation. An agar plate containing Luria–Bertani (LB) broth was used to sustain *E. coli* cells. A colony of *E. coli* cells was removed from the agar plate and suspended in 5 mL of LB liquid media. Overnight at 37 °C, the cells were cultured in the medium on a shaker at 200 rpm. After that, fresh LB medium was used to dilute the cell suspension until it reached an optical density of 0.01 at 600 nm.

**Figure 4:**
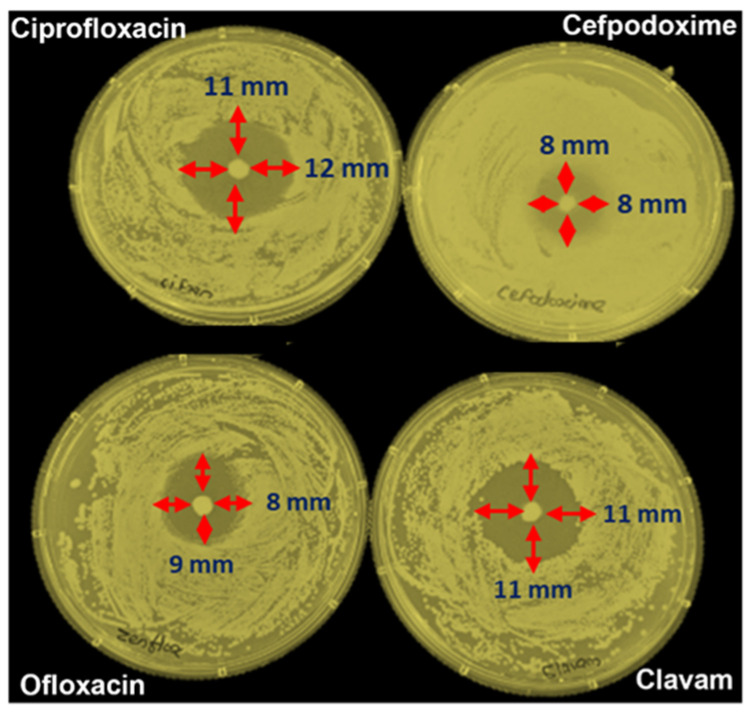
Disk diffusion method for calculation of minimum inhibitory concentration.

The antibiotic stock solution (1 mg/mL) of cefpodoxime, ofloxacin, amoxicillin, clavulanic acid, and ciprofloxacin was prepared in sterile water. Several concentrations, ranging from 100 to 500 g/mL, were prepared from the stock solution, and the MIC was measured using a disk diffusion approach. The LB agar media was prepared and poured into a Petri plate. After solidifying the agar gel, the bacterial culture was spread over the plate. Using sterile forceps, an antibiotic disk of cefpodoxime, ofloxacin, amoxicillin, clavulanic acid, and ciprofloxacin was applied to the plate and incubated for 12 to 24 h at 37 °C. The minimum inhibition zone formed at the edge of the antibiotic disk was calculated. Figure 4 shows the disk diffusion method for MIC calculation. The distance from the antibiotic disk to the inhibition area for every antibiotic was calculated. The one that covered more inhibition zones, i.e., ciprofloxacin, was selected for a real sample and interference study.

### 3.2. Electrochemical Detection of Antibiotic Effect over the Bacterial Growth

**Figure 5:**
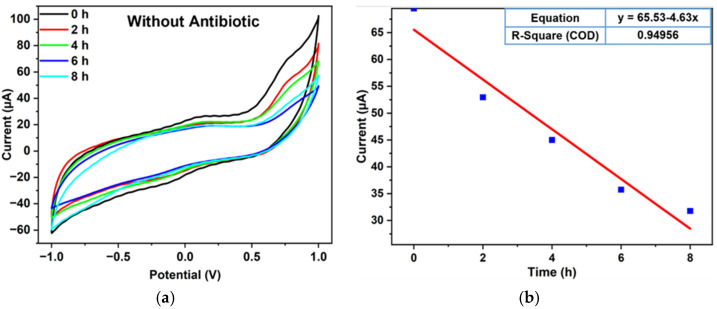
A cyclic voltammetric graph of bacterial culture without antibiotics (**a**) and its respective calibration plot (**b**).

**Figure 6:**
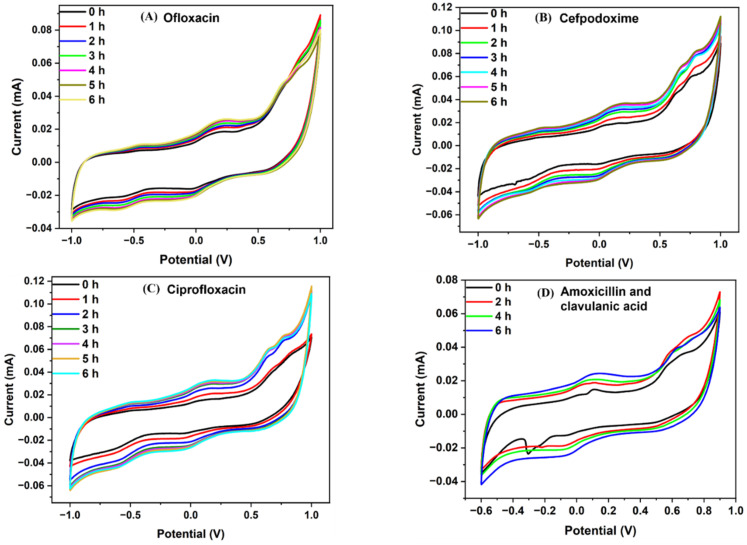
Cyclic voltammetric graphs of four antibiotics at concentrations of 100 µg/mL. The experiment was performed in the microfluidic device for 6 h, and the response was recorded at intervals every 1 h. (**A**) Ofloxacin, (**B**) cefpodoxime, (**C**) ciprofloxacin, (**D**) amoxicillin and clavulanic acid.

**Figure 7:**
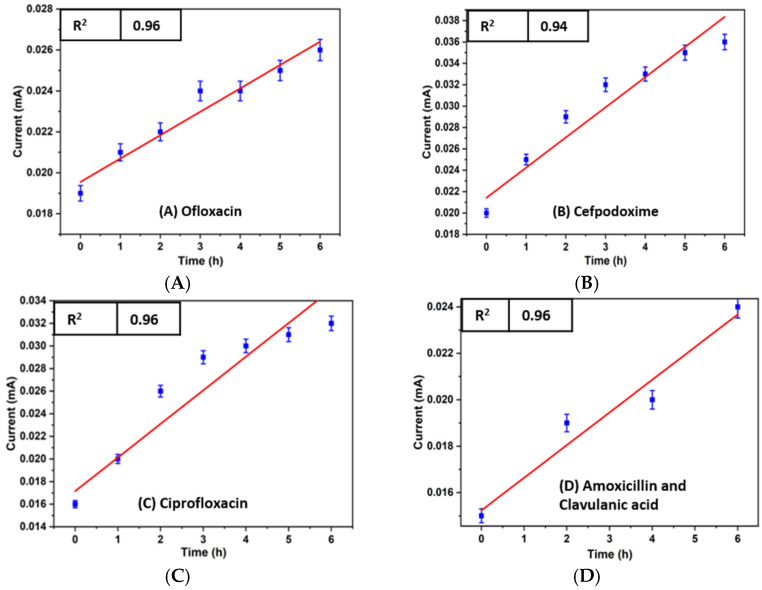
Calibration plot of four antibiotics was performed in the microfluidic device for 6 h. (**A**) Ofloxacin, (**B**) cefpodoxime, (**C**) ciprofloxacin, (**D**) amoxicillin and clavulanic acid.

To carry out the electrochemical investigation of bacterial growth inhibition, 100 µg/mL antibiotic concentration of cefpodoxime, amoxicillin and clavulanic acid, ciprofloxacin, and ofloxacin with bacterial culture media was injected into four miniaturized reservoirs through the inlet. Before the analysis, the reservoir was washed with 0.1 M PBS to prevent cross-contamination. The LIG heater was used to incubate the bacterial culture throughout the experiment, which is necessary for bacterial growth. The electrochemical detection was carried out using CV for 6 h, and after every hour, the response was recorded. Figure 5 and Figure 6 show the electrochemical reactions of the control sample (without antibiotic) and four antibiotics, and their respective calibration plots are given in Figure 7. According to the minimum inhibition zone study, out of four antibiotics, ciprofloxacin was more effective toward *E. coli* bacterial inhibition. The bacterial concentration would decline in the device with increased time and a constant temperature. In Figure 6, the current value increases with incubation time because of antibiotics on bacterial growth. Usually, when the bacteria grow, they accumulate over the electrode surface and block ion flow, decreasing the peak current value, which we can see in Figure 5 without antibiotic response. The antibiotic helps to increase the transfer of ions in the media, which was blocked by the growth of bacteria, as shown in Figure 6 [32].

### 3.3. Interference Study

**Figure 8:**
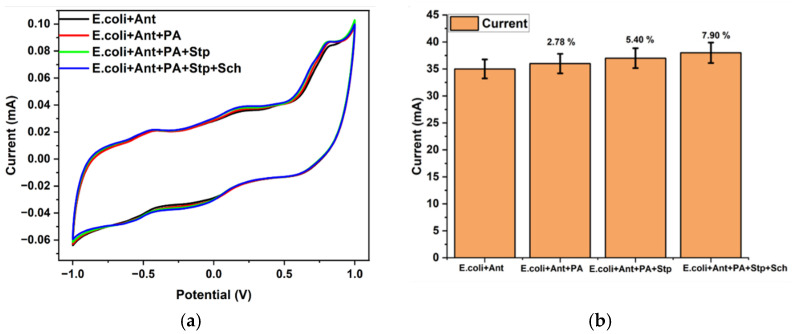
CV graph for specificity study of antibiotics (Ant) toward *E. coli*, *Pseudomonas aeruginosa* (PA), *Streptococcus pneumoniae* (Stp), and *Shewanella putrefaciens* (Sch). CV graph responses (**a**) and respective total current plot variation (**b**).

The specificity of the Clavam antibiotic toward *E. coli* was checked in the developed microfluidic device. Four variants, namely *Streptococcus*, *Shewanella*, *Pseudomonas*, and *E. coli*, were tested with Clavam antibiotic. The first *E. coli* with Clavam antibiotic was injected into the device, and CV response was recorded, Figure 8a. *Pseudomonas*, *Streptococcus*, and *Shewanella* were then added into the same device, and the CV response was measured. A similar current histogram for *E. coli* and an additional variant mixed with *E. coli* is shown in Figure 8b. The apparent difference in the inhibition values for *E. coli* and other bacterial species confirms that ciprofloxacin did not affect *Shewanella putrefaciens*, *Pseudomonas aeruginosa*, and *Streptococcus pneumoniae* [25]. The efficiency of ciprofloxacin against different pathogens was negligible or less than 10%, indicating that it is solely effective against *E. coli* species or that the developed sensor is specific toward *E. coli* species.

### 3.4. Antibiotic Susceptibility Testing Using Synthetic Urine

A urine sample is mainly used for examining a medical condition. However, obtaining the same quality urine for many illness detections takes time and effort. Hence, synthetic urine is used for experimentation purposes [33]. Along with this, water pollution due to microorganisms is also a significant issue that causes waterborne diseases. Therefore, there is a critical need to detect the microbial pollution of water [34]. Consequently, urine and water samples were selected for real sample analysis. Before testing, synthetic urine and tap water were autoclaved to avoid any microbial contamination.

**Figure 9:**
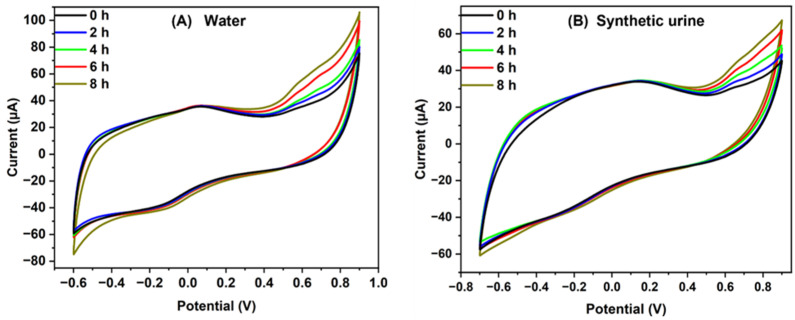
CV graph for real sample analysis performed in the microfluidic device for 7 h in (**A**) tap water and (**B**) synthetic urine.

*E. coli* culture was inoculated into the synthetic urine and tap water before being injected into the device. The electrochemical response was checked for 7 h at hourly intervals. The CV response of a water sample and synthetic urine is shown in Figure 9. The rise in current under the influence of antibiotics was observed [35]. As time increases, the current values also increase because the antibiotic decreases the growth of bacteria. The volume of ions in the urine increases the flow of ions, increasing the current value. This signifies the effect of antibiotics on the growth of bacteria.

## 4. Conclusions

This study developed a label-free, simple, cost-effective, miniaturized electrochemical microfluidic device to diagnose microbial resistance and bacterial infection rapidly. The designed device demonstrated remarkable sensitivity toward *E. coli* and detected the antibiotic susceptibility within a concise 6-h timeframe. As part of our methodology, a three-electrode system was added to our miniaturized platform, with the working electrode modified with GMC, the reference electrode using Ag/AgCl, and the counter electrode composed of bare carbon ink. Electrochemical detection was performed using cyclic voltammetry, which enabled accurate measurements. The working electrode was modified with GMC to improve sensor performance and increase precision. Four antibiotics of the same concentration were tested in the susceptibility study, and the interference of the antibiotic toward *E. coli* was detected. Further antibiotic susceptibility study was performed in both artificial urine and water samples, ensuring the versatility and robustness of our device’s performance. The obtained outcomes were validated with the conventional approach, confirming the reliability of our findings. To address the pressing demand for rapid and accurate antibiotic susceptibility testing, integration with Cyber-Physical System (CPS) augmented techniques, like data mining and machine learning, with essential automation will aid in diagnosing antibiotic susceptibility rapidly. These technologies will advance the diagnostic process and enhance its accuracy. Moreover, as bacterial resistance to conventional treatments continues to rise, there is a critical need for technologies that accurately distinguish between resistant, susceptible, and persistent bacterial strains. These advancements pave the way for precision medicine, yielding optimal diagnostic results in the ever-evolving landscape of bacterial infections.

## Figures and Tables

**Table 1:** Composition of synthetic urine.

Components	Quantity (mg/L)
Potassium Chloride (KCl)	2000
Sodium Sulfate (Na_2_SO_4_)	2000
Ammonium Phosphate ((NH_4_)_3_PO_4_)	850
Ammonium Diphosphate ((NH_4_)_3_PO_4_)	850
Calcium Chloride (CaCl_2_)	250
Magnesium Chloride (MgCl_2_)	500
Urea (CH_4_N_2_O)	600
Creatinine (C_4_H_7_N_3_O)	50

## Data Availability

No new data were created or analyzed in this study. Data sharing is not applicable to this article.
